# Supplementary material for: Content-rich biological network constructed by mining PubMed abstracts
Source: BMC Bioinformatics. 2004 Oct 8;5:147. doi: 10.1186/1471-2105-5-147 (PMC528731; doi:10.1186/1471-2105-5-147)
Supplement: Additional File 2 — The original results of the above study (non-essential files are deleted to keep the file size under the limit set by BMC bioinformatics). [file 1471-2105-5-147-S2.bz2 › chilibotAdditionalFile2/dip10/566ID10574949E10927/html/FGFR3_FGF9.html]

 


 **FGFR3** and **FGF9** 
  
Found 19 abstracts in PubMed, retrieved 10.  
 

 What does Google say? 
 PDF only 
| .edu only 

---

**Interactive relationship** (e.g. stimulation, inhibition, etc)

**Neutral relationship**- Since receptor activation can result in its movement to a perinuclear localization, an alternative explanation for the redistribution of FGFR 3  [ **FGFR3** ]  IIIb could be different degrees of activation by a ligand FGF1 or  **FGF9** .  Ref: 11329138 J Pathol, 2001

**Non-interactive relationship** (e.g. studied together, co-existance, homology, etc.)

- Moreover, a model is presented providing a molecular basis for the preferential affinity of  **FGF9**  toward  **FGFR3** .  Ref: 11060292 J Biol Chem, 2001
- Expression at the mRNA level of  **FGF9** ,  **FGFR3** , and collagen type IIa is found in these cells, but not in skin fibroblasts from afflicted or healthy individuals.  Ref: 11518409 Orthopedics, 2001
- Expression of each FGFR and effects of the preferred ligand for FGFR 3  [ **FGFR3** ] ,  **FGF9** , upon RGC survival and neurite outgrowth were examined in primary retinal cell cultures whereas there was no stimulation of neuritogenesis, RGC survival was promoted in a dose dependent manner ED 50 approximately 500 pg ml, mean maximal increase of 60% and could be completely blocked by addition of  **FGF9**  neutralising antibody.  Ref: 12799136 Mol Cell Neurosci, 2003
- Expression patterns of  **FGFR3**  and its specific ligand, fibroblast growth factor 9  [ **FGF9** ]  FGF 9  [ **FGF9** ] , were evaluated both in situ and in cell cultures.  Ref: 10971739 Int J Exp Pathol, 2000
- Taken together, these data suggest that the ligand receptor couple  **FGF9**  FGFR 3  [ **FGFR3** ]  may function to promote survival of adult mammalian RGC, and their application might be beneficial in retinal degenerative diseases such as glaucoma.  Ref: 12799136 Mol Cell Neurosci, 2003
